# Estrogen Regulates the Tumour Suppressor MiRNA-30c and Its Target Gene, MTA-1, in Endometrial Cancer

**DOI:** 10.1371/journal.pone.0090810

**Published:** 2014-03-03

**Authors:** Xiangyi Kong, XiaoFeng Xu, Yuhua Yan, Feifei Guo, Jian Li, Yali Hu, Huaijun Zhou, Qingying Xun

**Affiliations:** 1 Department of Gynecology and Obstetrics, Nanjing Drum Tower Hospital Affiliated to Nanjing University Medical School, Nanjing, China; 2 Department of Physiology, Medical College, Southeast University, Nanjing, China; 3 Reproductive Medicine Center, Nanjing Drum Tower Hospital Affiliated to Nanjing University Medical School, Nanjing, China; Roswell Park Cancer Institute, United States of America

## Abstract

MicroRNA-30c (miR-30c) has been reported to be a tumour suppressor in endometrial cancer (EC). We demonstrate that miR-30c is down-regulated in EC tissue and is highly expressed in estrogen receptor (ER)-negative HEC-1-B cells. MiR-30c directly inhibits MTA-1 expression and functions as a tumour suppressor via the miR-30c-MTA-1 signalling pathway. Furthermore, miR-30c is decreased upon E_2_ treatment in both ER-positive Ishikawa and ER-negative HEC-1-B cells. Taken together, our results suggest that miR-30c is an important deregulated miRNA in EC and might serve as a potential biomarker and novel therapeutic target for EC.

## Introduction

Endometrial cancer (EC) is the most frequent malignant tumour of the female genital tract worldwide. Endometrial cancer comprises two different pathogenetic subtypes. Type I tumours are low-grade estrogen-related endometrioid cancers (EEC) that generally develop from complex and atypical endometrial hyperplasias in peri-menopausal women. In contrast, type II tumours are aggressive non-endometrioid cancers (NEEC) that are unrelated to estrogen stimulation and that develop from the atrophic endometrium of elderly women. The differences between the two EC subtypes lead to different treatment and prognoses[1].

The importance of microRNAs (miRNAs), one type of the non-coding RNA, has been demonstrated in cancer. Genes encoding mammalian miRNAs are initially transcribed as primary miRNAs (pri-miRNAs), which are processed by the enzymatic complexes Drosha and Dicer to become precursor miRNAs (pre-miRNAs) and mature miRNAs[2]. The mature miRNAs are approximately 22-nucleotide-long, single-stranded RNA molecules that regulate the expression of their target genes by imprecise complementation to the 3′-untranslated regions (UTRs), 5′-UTRs, and even coding sequences of the mRNAs to repress translation in animals[2]–[5]. Estrogen (E_2_) and the estrogen receptor (ER) have been shown to modulate miRNAs such as miR-125a[6] and miR-429[7] in mouse uterus, miRNA-20a and miRNA-21 in normal endometrial glandular epithelial cells[8], Let-7 family, miR-27a, miR320 and miR-424[9] in EC cells, miR-104[10], miR-7[11], miR-21[12], miR-30c and miR-103[13] in breast cancer cells, miR-135b in colorectal cells[14] and miR-203 in vascular smooth muscle cells[15]. Taken together, these results indicate that E_2_ and ER play important roles in miRNA regulation.

Recently, the deregulation of miR-30c has been reported not only to be relevant to the tumorigenesis and progression of many cancers, such as EC[16], [17], ovarian cancer [18], [19], breast cancer[20]–[23], lung cancer[24], clear cell renal cell carcinoma[25], gastric cancer[26], bladder cancer[27] and neuroblastoma[28], but also to exhibit potential diagnostic and prognostic implications, as well as to represent a potential therapeutic target of chemo- and radiotherapies for cancers[18], [29], [30]. Currently, metastasis-associated gene-1 (MTA-1)[17], KRAS[20], DLL4[31], TWF1, vimentin[21], BCL9[19], REDD1[32], PAI-1[33] and CTGF[34] have been identified as targets of miR-30c, and SERPINE1[28], NYH11, GPRASP2 DDR2[16], PPARGC1B, Makorin-3, UBAC1, PTPDC1[22] snail1[35], p53[36]are potential targets that remain to be validated. Because miR-30c exhibits a relative decrease in expression from normal endometrium to atypical hyperplasia to cancer, and because the role of miR-30c in EC remains unknown, we conducted an investigation of the role of miR-30c in EC. Our previous studies have found that miR-30c targets MTA-1, which is highly expressed in EC[37] and functions as tumour suppressor in EC cell lines[17]. However, the precise roles of miR-30c and MTA-1 in EC are unclear. Therefore, we conducted this extended study.

In this study, we evaluated the expression of miR-30c in EC tissues and different EC cell lines, further investigated the relationship between miR-30c and MTA-1, validated the tumour suppressor function of miR-30c and explored the regulation of miR-30c in EC cell lines. Thus, we sought to determine the tumour suppression function of miR-30c, which would define its potential value as a novel therapeutic target for the treatment of EC.

## Materials and Methods

### Patients and tissue samples

This study was approved by the Ethics Committee of Nanjing Drum Tower Hospital Affiliated to Nanjing University. Study subjects were recruited from the patients at the Department of Obstetrics and Gynecology, Nanjing Drum Tower Hospital, Nanjing University Medical School in Nanjing, China. Written informed consent was received from all participants involved in the study. All of samples were collected with patients' informed consent and confirmed by the pathological examination. All the patients were suffering type I EC and none of them received preoperative treatment, such as radiation therapy or chemotherapy. 21 primary EC tumour tissue specimens were obtained from the EC patients, and 14 normal endometrial tissue samples were obtained from women who underwent hysterectomies (laparoscopic or abdominal) for the treatment of other diseases such as myoma or uterine prolapse.

### EC cell lines and treatments

Human EC Ishikawa cells were kindly provided by Professor L.H.Wei (Peking University People's Hospital, China), and HEC-1-B were purchased from the Shanghai Cell Collection (Shanghai, China). The Ishikawa cells were cultured in Dulbecco's Modified Eagle's Medium (DMEM, Gibco, USA), and the HEC-1-B cells were cultured in McCoy's 5A Medium (Gibco, USA), both of which were supplemented with 10% fetal bovine serum (FBS, Gibco). The cells were maintained in a 5% CO_2_ humidified atmosphere at 37°C. To determine the regulation of E_2_, cells were treated with 17β-estradiol (E_2_, 10^−8^ M and 10^−10^ M, Sigma, USA) for 6, 12, 24 and 48 h in 6-well plates. Ishikawa and HEC-1-B cells were co-treated with the ER antagonist Fulvestrant (ICI 182780, 10^−8^ M, Sigma, USA) and 10^−8^ M or 10^−10^ M E_2_ for 24 and 48 h, respectively.

### Cell transfection

The mimics (miR10000244) and inhibitor (miR20000244) of miR-30c, small interfering RNA of MTA-1 (si-MTA-1, si-h-MTA1_001) and their respective scramble oligonucleotides, mimics-sc (miR01101), inhibitor-sc (miR02101), siR-sc (siN05815122147), were designed and synthesised by Guangzhou RiboBio (Guangzhou, China). All of the oligonucleotides were transfected into cells using Lipofectamine^TM^ 2000 (Invitrogen, USA) in antibiotic-free Opti-MEM medium (Invitrogen, USA) according to the manufacturer's protocol at a final concentration of 50 nM. For the cotransfections, 25 nM of each oligonucleotide was used. Total RNA and proteins were extracted at 48 h post-transfection for further analysis. Non-transfected Ishikawa cells in culture medium were also prepared to serve as mock controls.

### Quantitative real-time polymerase chain reaction (qRT-PCR)

Total RNA of tissue and cells was extracted using TRIzol reagent (Invitrogen, USA). The stem-loop RT primer of miR-30c, primers of miR-30c, U6 and MTA-1 for qRT-PCR were described in our previous study[17]. The GAPDH, pri-miR-30c, and pre-miR-30c primers were designed as follows: GAPDH forward primer: TGAACGGGAAGCTCACTGG, GAPDH reverse primer: TCCACCACCCTGTTGCTGTA; pri-miR-30c forward primer: GCCCAAGTGGTTCTGTGTTT, pre-miR-30c forward primer: ACCATGCTGTAGTGTGTGTAAACA, and pri-miR-30c/pre-miR-30c reverse primer: TCCATGGCAGAAGGAGTAAA. cDNA was synthesised from total RNA by reverse transcription using the PrimeScript™ RT reagent Kit (TakaRa, Dalian, China). Next, qRT-PCR was performed using the SYBR PrimeScript™ RT-PCR Kit (TakaRa, Dalian, China) according to the manufacturer's protocol. The relative expression levels of miR-30c and MTA-1 were determined using the 2^−ΔΔCt^ analysis method; the levels of GAPDH and U6 were used as internal controls for MTA-1 and miR-30c, pri-miR-30c, pre-miR-30c.

### Western Blot

Cells were harvested and lysed in radioimmunoprecipitation assay (RIPA) lysis buffer (Sigma, USA) containing a protease inhibitor cocktail (Roche Diagnostics, Mannheim, Germany). The protein concentrations of the total cellular lysates were measured using the Micro BCA protein assay kit (Pierce Biotechnology Inc., Rockford, USA). An equal amount (50 µg) of each cellular lysate was resolved by electrophoresis in 10% SDS-polyacrylamide gels (SDS-PAGE) and transferred onto polyvinylidene fluoride (PVDF) membranes (Millipore, Billerica, MA), which were blocked in TBST with 10% non-fat, dried milk. The membranes were probed with a primary antibody against MTA-1 (1∶500, Abcam, Cambridge, UK) and a secondary horseradish peroxidase (HRP)-conjugated antibody (1∶5000, Bioworld Technology, USA). The proteins of interest were then detected using an enhanced chemiluminescence (ECL) blotting detection system (Millipore, Billerica, MA). GAPDH was used as a loading control. The relative intensity of the target bands was analysed using Quantity One.

### Cell proliferation assay

Cell proliferation was analysed using a 3-(4,5-dimethylthiazol-2-yl)-2,5-diphenyltetrazolium bromide (MTT) assay. Cells were seeded into 96-well plates (5×10^3^ cells/well) directly or at 24 h after transfection and incubated for 24, 48, 72 and 96 h, respectively. After incubation with 25 µl of MTT (5 mg/ml, Sigma, USA) at 37°C for 4 h, the supernatants were removed, and 150 µl of dimethylsulfoxide (DMSO, Sigma, USA) was added to each well. The absorbance value (OD) of each well was measured at 490 nm. For each experimental condition, 6 wells were used, and the experiment was performed in triplicate.

### Cell migration and invasion assay

Cell migration was measured using a wound-healing assay. The transfected cells were seeded into 6-well plates and cultured to confluence. Wounds were made using a p10 pipette tip, and the cells were washed with PBS to clear debris and detached cells. The cells were then incubated in serum-free medium for another 24 or 48 h. The individual gaps were observed and photographed using an inverted microscope at 0, 24 and 48 h at the same position of the wound.

Cell invasion assays were performed in 24-well, Matrigel-coated invasion chambers. At 48 h post-transfection, 2×10^4^ cells in 0.2 ml serum-free-DMEM were added to the upper chambers (8-µm, Millipore), which were coated with 30 µl matrigel (BD Bioscience, San Jose, CA), and 0.6 ml of 10% FBS-DMEM was added as the chemoattractant to the lower chamber. The cells were incubated at 37°C for 24 h, after which, the non-invading cells were removed with cotton swabs. The invading cells were stained with 0.1% crystal violet. The cells were counted in 5 random high-power fields at ×200 magnification per well. The experiment was performed in triplicate.

### Statistical analysis

Statistical analyses were performed using SPSS 19.0 software (SPSS, Chicago, USA). The values are presented as the means ± SD. Student's t-test was used to compare the values of the test and control samples. Differences between treatment groups were examined for statistical significance using one-way ANOVA. The statistical significance level was designated as P<0.05.

## Results

### Expression of MiR-30c and MTA-1 in EC samples and cell lines

**Figure 1:**
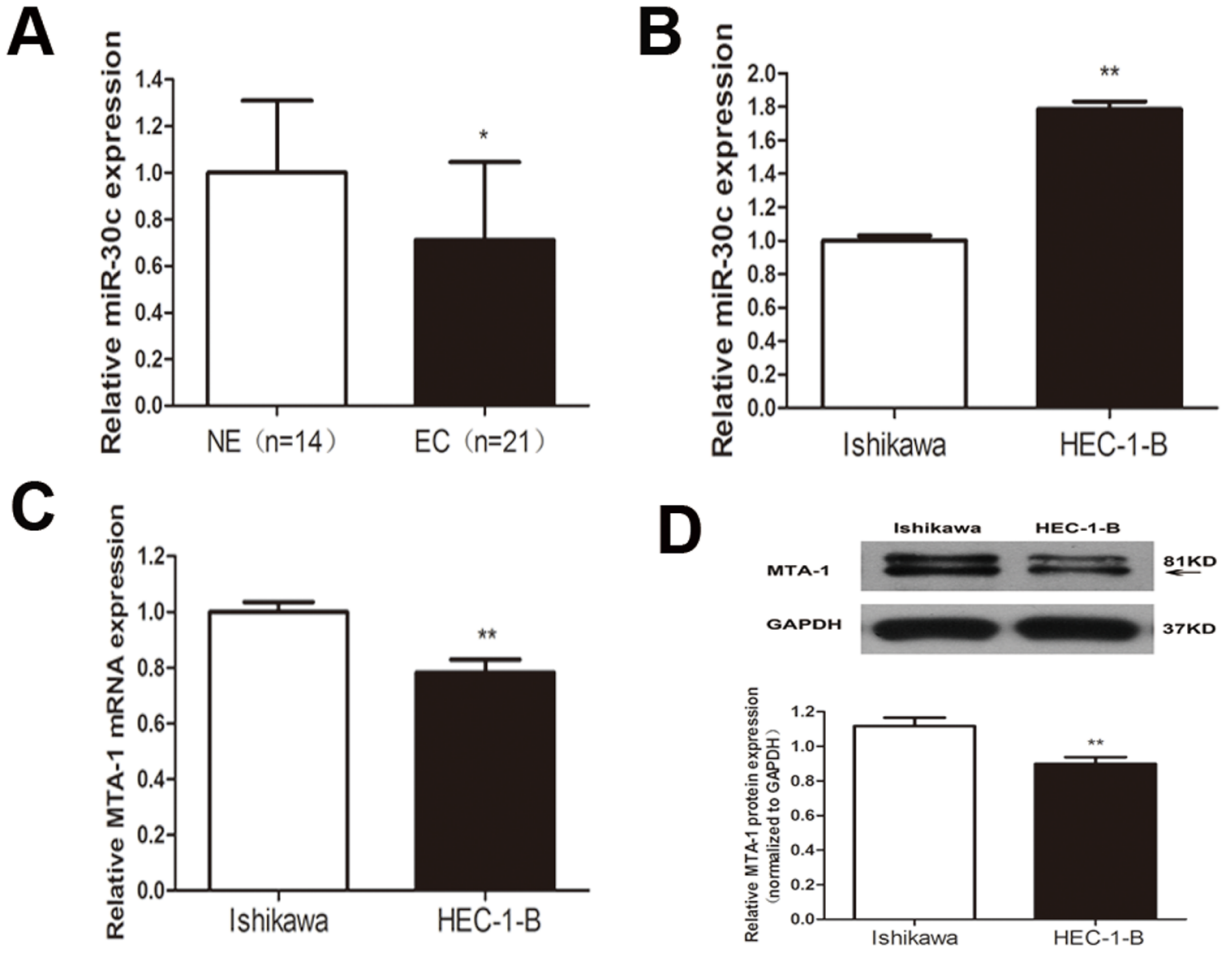
The expression of miR-30c and MTA-1 in EC samples and cell lines. (A). MiR-30c was decreased in 21 EC samples compared with 14 NE samples, as indicated by qRT-PCR analysis. Every sample was evaluated in triplicate. (B). MiR-30c was highly expressed in HEC-1-B cells compared with Ishikawa cells, as indicated by qRT-PCR analysis. (C) and (D). The expression of MTA-1 was decreased in HEC-1-B cells compared with Ishikawa cells at the mRNA (C) and protein (D) levels. Each bar represents the mean values ± SD from three independent experiments. (*P<0.05,**P<0.01).

MiR-30c was reported to exhibit relatively decreased expression from normal endometrial to atypical hyperplasia to cancer[16] and to function as a tumour suppressor in EC cell lines[17]. However, no other studies have demonstrated that miR-30c plays a role in EC. Therefore, we first investigated the relative expression of miR-30c among the tissue samples in this study. Our qRT-PCR analyses showed that miR-30c is significantly decreased in EC samples (Fig 1A.), suggesting that miR-30c plays a role in the incidence of EC.

We next evaluated the expression of miR-30c in different cell lines. ER-positive Ishikawa cells and ER-negative HEC-1-B cells represent models of type I and type II EC, respectively. Our qRT-PCR analysis revealed that miR-30c is highly expressed in HEC-1-B cells compared with Ishikawa cells, by 1.79-fold (Fig 1B.), suggesting that the expression of miR-30c correlates with ER or E_2_. Next, we assessed the levels of MTA-1 expression in different cell lines and found that it is highly expressed in Ishikawa cells at both mRNA and protein levels (Fig 1C and 1D). Taken together, our results indicate a negative correlation between miR-30c and MTA-1, consistent with our previous conclusion that miR-30c targets MTA-1.

### Variation of miR-30c expression modulates the expression of its target gene, MTA-1, in EC cells

We previously demonstrated that miR-30c targets MTA-1 mRNA transcripts in the Ishikawa and HEC-1-B cell lines using a Luciferase reporter assay. To thoroughly elucidate the relationship between miR-30c and MTA-1, we used miR-30c-mimics and miR-30c-inhibitor to restore and to reduce the expression of miR-30c, respectively. In addition, we used siR-MTA-1 to knockdown MTA-1 in Ishikawa cells.

**Figure 2:**
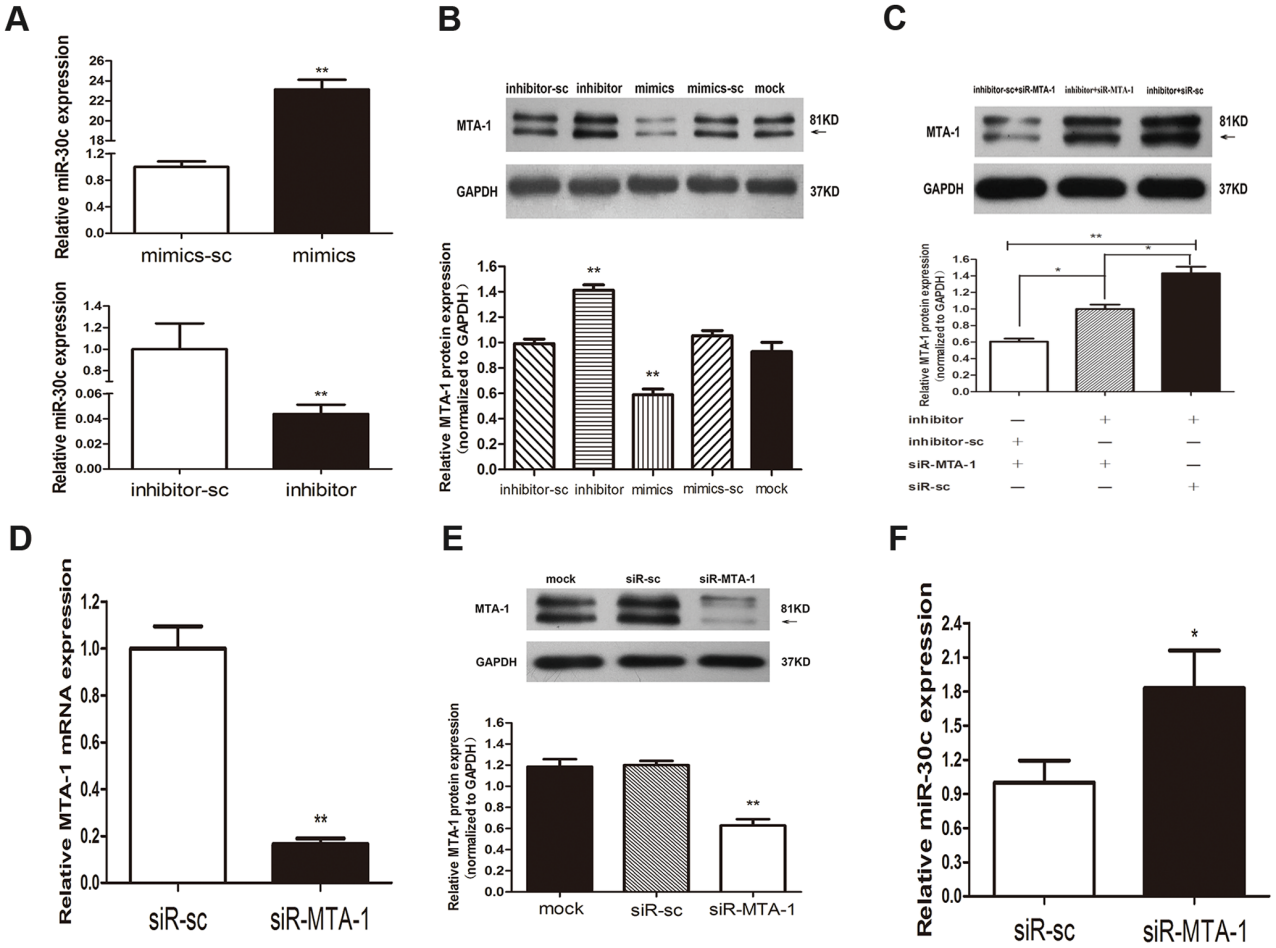
Variation of miR-30c expression modulates the expression of its target gene, MTA-1, in EC cells. (A). The transfection efficiency of Ishikawa cells was evaluated by qRT-PCR at 48 h after transfection of miR-30c-mimics and miR-30c-inhibitor, shown as fold-changes relative to their scrambled controls. (B). MTA-1 protein expression was assessed by western blot analysis at 48 h after transfection with miR-30c-mimics, miR-30c-inhibitor and their scrambled controls. (C). Cotransfection with miR-30c inhibitor, siR-MTA-1 and their scrambled controls was performed, and MTA-1 protein expression was assessed by western blot analysis. (D and E). SiR-MTA-1 reduces MTA-1 expression at the mRNA (D) and protein (E) levels at 48 h after transfection. (F). QRT-PCR analysis shows an increase in miR-30c expression after MTA-1 expression was inhibited. Each bar represents the mean values ± SD from three independent experiments. (*P<0.05, **P<0.01).

The expression of miR-30c in Ishikawa cells was up-regulated by 23.14-fold and down-regulated by 0.043-fold by transfection with miR-30c-mimics and miR-30c-inhibitor, respectively (Fig 2A). Upon sufficient transfection efficiency, we found that MTA-1 expression was significantly decreased when miR-30c was restored and that the reduction of miR-30c up-regulated MTA-1 expression (Fig 2B).

In cotransfection experiments, we found that miR-30c inhibitor and siR-MTA-1 played an antagonistic manner in MTA-1 regulation (Fig 2C). Together with the observation that siR-MTA-1 suppresseed the expression of MTA-1 at both the mRNA and protein levels (Fig 2D and 2E), we believe that miR-30c does in fact negatively regulated MTA-1. Interestingly, the repression of MTA-1 expression by siR-MTA-1 resulted in an increased expression of miR-30c (Fig 2 F).

Taken together, we confirmed that miR-30c directly repressed MTA-1 expression and that a feedback-loop is present between them. Although more studies are needed to explore the specific mechanism underlying their feedback-loop regulation, these results supported the notion that miR-30c might work by inhibiting MTA-1 in EC.

### MiR-30c regulates cell proliferation, migration and invasion in EC

**Figure 3:**
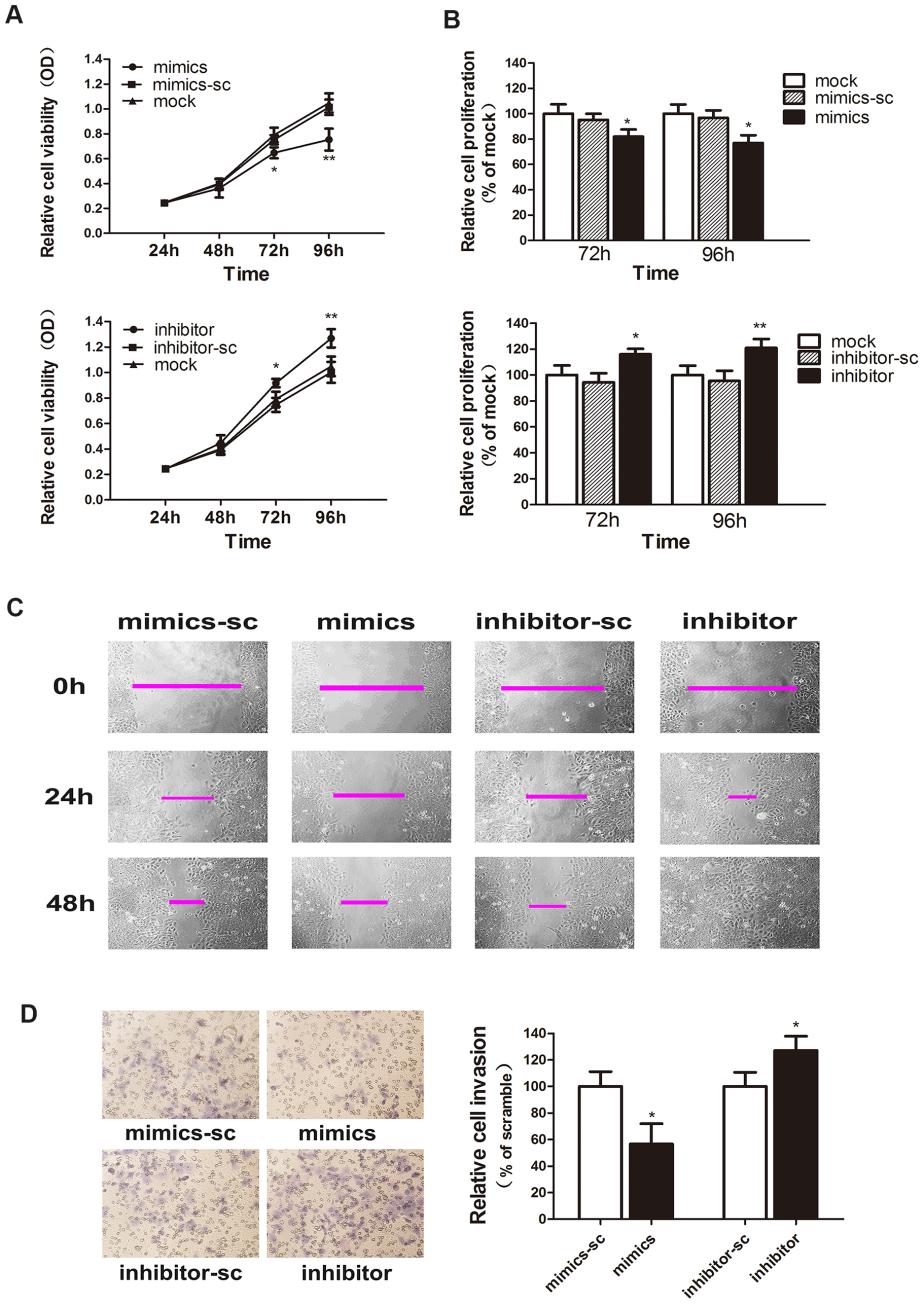
MiR-30c regulates cell proliferation, migration and invasion in EC. (A). A MTT assay shows that miR-30c mimics suppressed cell viability (upper), whereas miR-30c inhibitor promoted cell viability (lower). (B). MiR-30c-mimics reduces relative cell proliferation (upper), whereas miR-30c-inhibitor increases relative cell proliferation (lower), respectively at 72 and 96 h after transfection. (C). Representative photographs of the wound-healing assay showing the migratory ability of the transfected cells at 0, 24 and 48 h after wounding. (D). Representative photographs of cell invasion in a transwell assay. The average number of cells was counted from 5 random microscopic fields (×200). The values shown are the mean values ± SD of relative cell invasion. (*P<0.05, **P<0.01).

As our previous study has shown, the ectopic expression of miR-30c can inhibit EC cell proliferation, migration and invasion[17]. Here, we provided further evidence to confirm these effects in Ishikawa cells. The ectopic expression of miR-30c inhibited cell proliferation in a MTT assay. The viability of transfected was repressed (Fig 3A) and relative cell proliferation was reduced at 72 and 96 h post-transfection (Fig 3B). In terms of migration and invasion, we performed a wound healing and a transwell assay. Transfection of miR-30c-mimics decreased migration and invasion compared with the mimics-sc, as shown in Fig 3C and 3D. On the other hand, the abilities of cell proliferation, migration and invasion were enhanced following the cells were transfected with miR-30c-inhibitor (Fig 3A -3D).

Thus, variable expression of miR-30c elicited different effects on the malignant characteristics of Ishikawa cells. We believe that miR-30c acts as a tumour suppressor by influencing the proliferation, migration and invasion of EC cells.

### MiR-30c functions as a tumour suppressor via the miR-30c-MTA-1 signalling pathway

**Figure 4:**
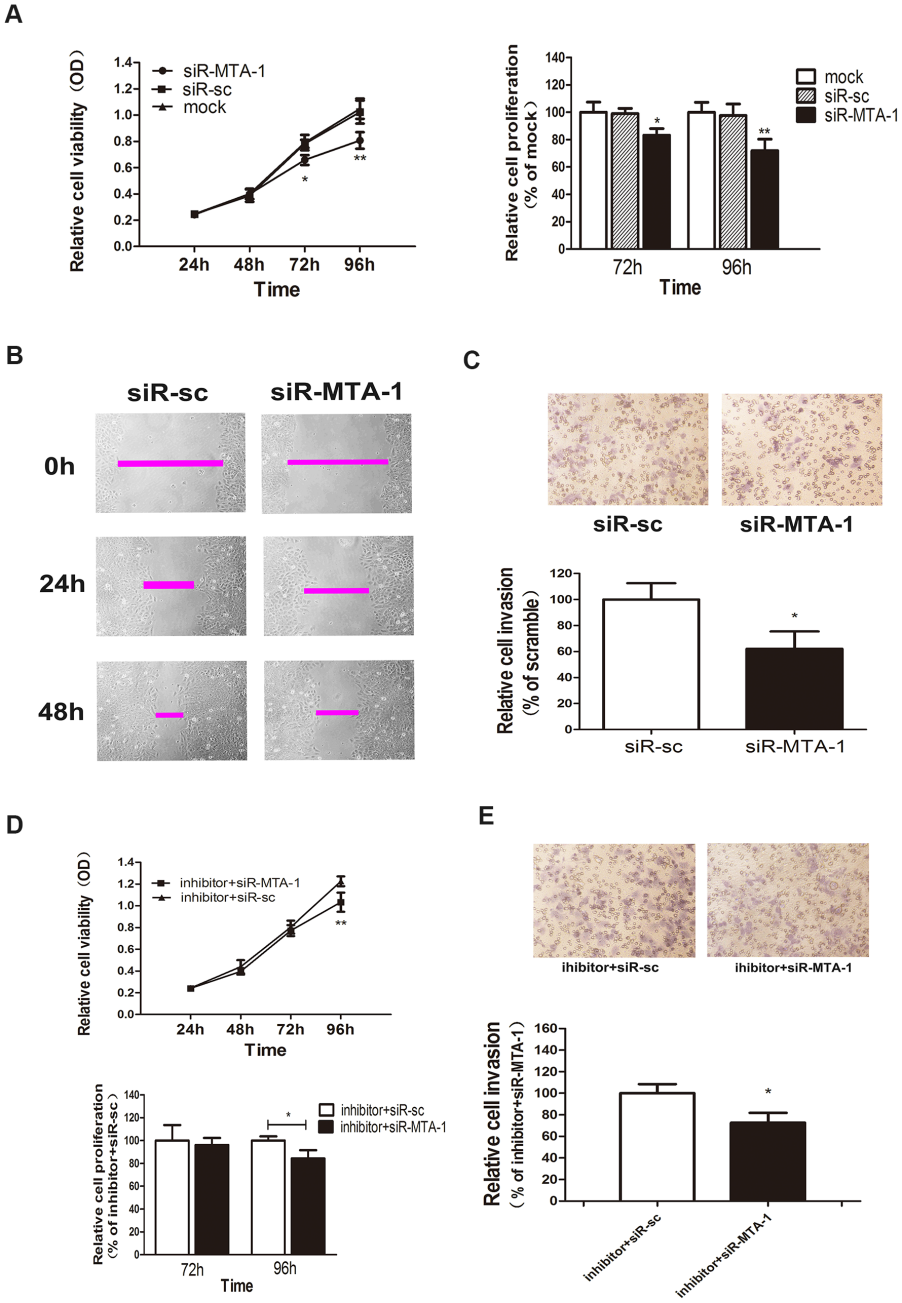
MiR-30c functions as a tumour suppressor through the miR-30c-MTA-1 signalling pathway. (A). A MTT assay shows the cell viability (left) and relative cell proliferation (right) were repressed by transfection of siR-MTA-1 at 72 and 96 h. (B). Representative photographs of the wound-healing assay show the migratory ability of the transfected cells at 0, 24 and 48 h after wounding. (C). Representative photographs of cell invasion in a transwell assay. The average number of cells was counted from 5 random microscopic fields (×200). The values shown are the mean values ± SD of relative cell invasion. (D) and (E). Cotransfection of miR-30c-inhibitor and siR-MTA-1; cotransfection of miR-30c-inhibitor and siR-sc served as the control. (D). Compared with the control transfection, cell viability (upper) and relative cell proliferation (lower) was suppressed at 96 h. (E). Representative photographs of cell invasion in a transwell assay. The average number of cells was counted from 5 random microscopic fields (×200). The values shown are the mean values ± SD of relative cell invasion. The differences of relative cell proliferation between the groups appeared at 96 h after transfection. (*P<0.05, **P<0.01).

We previously determined that miR-30c targets MTA-1, which is overexpressed in EC[37] and which acts as an oncogene in many human tumours [38], [39]. However, the function of MTA-1 in EC remained undefined. Therefore, we investigated whether miR-30c functions via the miR-30c-MTA-1 signalling pathway. Repression of MTA-1 by siR-MTA-1 inhibited the proliferation of Ishikawa cells, as indicated by our MTT results (Fig 4A). Moreover, loss of MTA-1 also reduced cell migration and invasion, as indicated by our wound healing and transwell assay results (Fig 4B and 4C). These findings revealed that MTA-1 plays a pro-tumourigenic role in EC cells by regulating the proliferation, migration and invasion of Ishikawa cells. Because miR-30c can directly repress the expression of MTA-1, and because MTA-1 is an oncogene in EC, we believe that miR-30c functions as a tumour suppressor by targeting MTA-1.

To extend our study, we cotransfected miR-30c-inhibitor and siR-MTA-1 into Ishikawa cells and found that the reduction of MTA-1 attenuated cell proliferation and invasion mediated by miR-30c-inhibitor compared with the control-transfected group (Fig 4D and 4E). Our results suggest that miR-30c function depends on MTA-1 and validated the existence of miR-30c-MTA-1 signalling pathway.

### Estrogen down-regulates expression of miR-30c in EC cells

Having demonstrated the tumour suppressive ability of miR-30c, we next investigated the regulation of miR-30c. The difference in miR-30c expression between ER-positive Ishikawa cells and ER-negative HEC-1-B cells suggests that E_2_ and ER might play important roles in the regulation of miR-30c. Thus, we investigated whether miR-30c expression changes upon E_2_ treatment.

**Figure 5:**
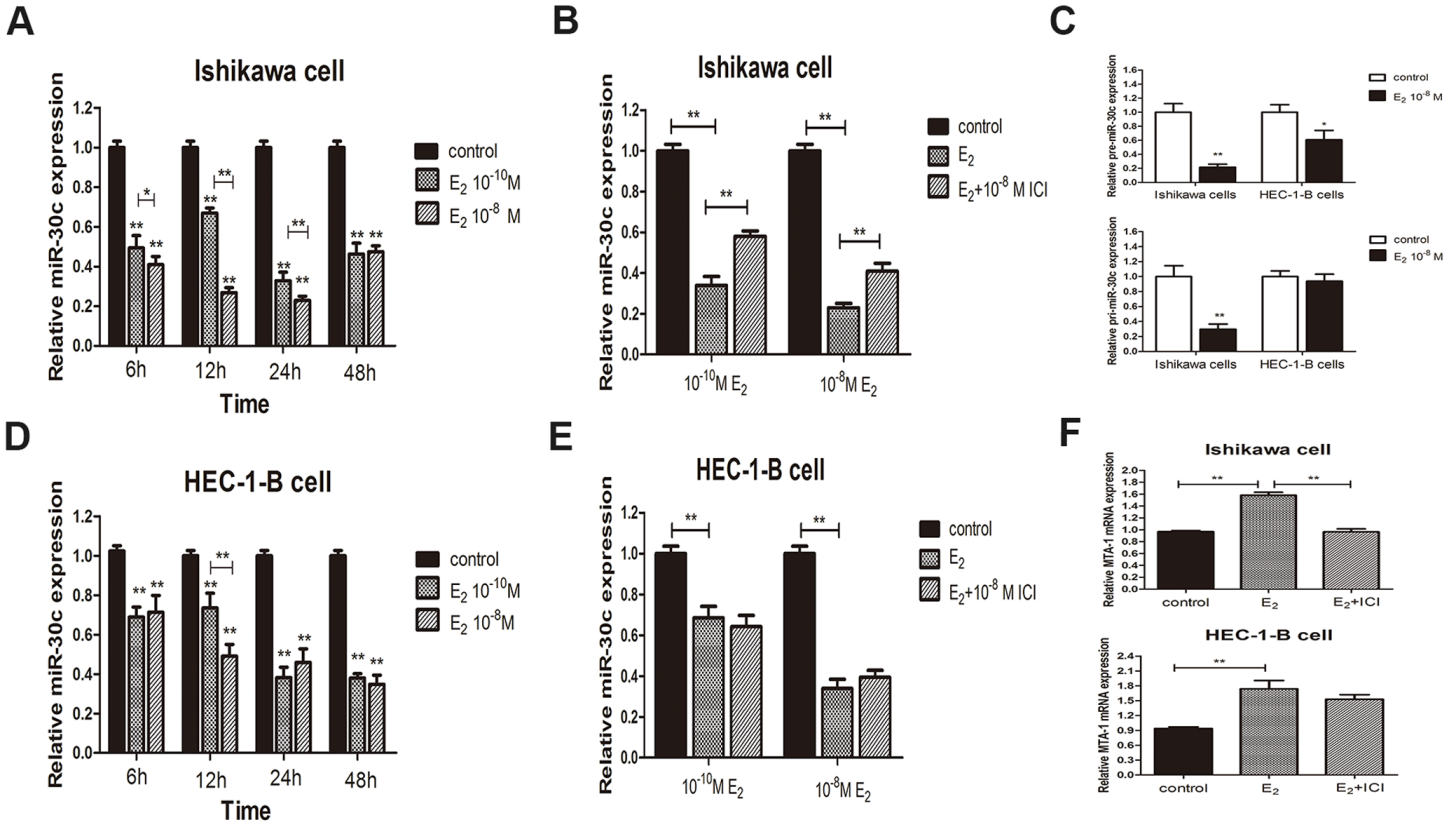
Estrogen regulates the expression of miR-30c in EC cells. (A). MiR-30c was decreased upon treatment with 10^−8^ M and 10^−10^ M E_2_ at 6, 12, 24 and 48 h after treatment by qRT-PCR analysis in Ishikawa cells. The treatment with 10^−8^ M E_2_ exerted a more significant inhibition except at 48 h. (B). 10^−8^ M ICI182780 partially reversed the reduction of miR-30c levels induced by the treatment with 10^−8^ M and 10^−10^ M E_2_ at 24 h after cotreatment in Ishikawa cells. (C). Pre-miR-30c levels were reduced by E_2_ treatment in both the Ishikawa and HEC-1-B cells (upper). Pri-miR-30c levels were reduced by E_2_ treatment in Ishikawa but not in HEC-1-B cells (lower). (D). MiR-30c levels were reduced upon 10^−8^ M and 10^−10^ M E_2_ treatment at 6, 12, 24 and 48 h after treatment by qRT-PCR analysis in HEC-1-B cells. The effect of the two concentrations differed at 12 h after treatment. (E). 10^-8^ M ICI182780 did not reverse reduction of miR-30c levels induced by the treatment with 10^−8^ M and 10^−10^ M E_2_ at 48 h after cotreatment in HEC-1-B cells. (F). Treatment with 10^−8^ M E_2_ up-regulated mRNA expression of MTA-1 in both the Ishikawa cells and HEC-1 cells at 24 and 48 h, respectively, an effect that was reversed by cotreatment with 10^−8^ M ICI182780 in the Ishikawa cells (upper) but not the HEC-1-B cells ((lower). (*P<0.05, **P<0.01).

Compared with the blank control, the expression of miR-30c was markedly down-regulated in a time- and dose-dependent manner at 6, 12, 24 and 48 h upon 10^-8^ M and 10^−10^ M E_2_ treatment of ER-positive Ishikawa cells (Fig 5A). The alterations in miR-30c expression induced by E_2_ treatment were in part reversed by cotreatment with ICI182780 in Ishikawa cells (Fig 5B). In addition to the E_2_ treatment-induced repression of miR-30c, pri-miR-30c and pre-miR-30c were also down-regulated (Fig 5C), suggesting that E_2_-ER can inhibit the transcription of miR-30c in the Ishikawa cells.

However, we found that E_2_ also reduced miR-30c expression levels in ER-negative HEC-1-B cells (Fig 5D). It was expectable that the inhibition of miR-30c expression by E_2_ treatment was not blocked by ICI182780 in HEC-1-B cells (Fig 5E). Different from Ishikawa cells, E_2_ only reduced the expression of pre-miR-30c but not pri-miR-30c in HEC-1-B cells (Fig 5C), suggesting that E_2_ should inhibit the maturation of miR-30c in an ER-independent manner.

In addition, E_2_ up-regulated the expression of MTA-1 mRNA in both Ishikawa and HEC-1-B cells, an effect that was reversed by ICI182780 in Ishikawa cells only at selected concentration and time (Fig 5F). This might be attributed to either the reduction of miR-30c induced by treatment with E_2_ or another as of yet unidentified signalling pathway.

Taken together, our findings indicate that E_2_ represents a regulatory factor for the expression of miR-30c, which functions in both ER-dependent and ER-independent manners. However, the precise underlying molecular mechanism by which E_2_ regulates the expression of miR-30c requires further detailed studies.

## Discussion

The deregulation of miRNAs in cancer can promote angiogenesis, growth advantage, tissue invasion and metastasis. However, our understanding of the aberrant expression and potential roles of miRNAs in cancer remains limited. MiR-30c, a member of the miR-30 family, has been shown to be down-regulated in EC in microarray analyses[16] and in breast cancer[23] compared with normal tissues but up-regulated in ovarian cancer compared with benign or borderline ovarian tumours[18] and in mesothelioma cells[29]. MiR-30c can also serve as a potential biomarker due to its deregulation in different subtypes and malignant stages of tumours including breast cancer [21], ovarian cancer[18] and mesothelioma[28], [29]. Furthermore, the increased expression of miR-30c correlates with favourable prognosis and clinical efficacy of tamoxifen therapy in breast cancer[22] as well as longer progression-free survival in clear cell renal carcinoma[25] but worse survival in malignant mesothelioma[29] and chemotherapeutic resistance in lung cancer[24]. Notably, in regards to the role of miR-30c in chemotherapeutic resistance in ovarian cancer, Eitan et al. and Sorrentino et al. have made contrasting conclusions[40], [41]. The controversial findings concerning miR-30c confirm its important role in tumourigenesis and progression, regardless of its precise tumourigenic or tumour suppressive nature.

In this study, we further investigated the role of miR-30c in EC. We found that miR-30c was down-regulated in EC samples compared with normal samples, as indicated by qRT-PCR analysis, consistent with the previous microarray analysis by Boren et al[16]. Unfortunately, we didn’t have enough cases to value the deregulation of miR-30c expression among different subtypes and clinicopathological features. Our future studies will aim to elucidate this issue. Nonetheless, we found that miR-30c expression was higher in an ER-negative cell line, implying that miR-30c might correlate with E_2_ and ER, as well as with specific subtypes of EC.

To extend our previous study, we thoroughly examined the relationship between miR-30c and MTA-1. Previously, we performed a Luciferase reporter assay that demonstrated the 3′-UTR of MTA-1 targeted by miR-30c. Furthermore, we showed that the over-expression of miR-30c decreased MTA-1 expression at the mRNA and protein levels in both Ishikawa and HEC-1-B cells[17]. Here, we not only restored but also reduced the expression of miR-30c and assessed the resulting changes in MTA-1. We used Ishikawa cells only in this study because miR-30c functioned the same in the two cells lines in our previous study. The results of this study are consistent with those of our previous study, and we also found that siR-MTA-1 and the miR-30c inhibitor worked in an antagonistic way. As we verified the direct repression of MTA-1 by siR-MTA-1, we were able to deduce a functional relationship between miR-30c and MTA-1. Remarkably, we also identified a feedback-loop wherein the repression of MTA-1 increased levels of miR-30c, a feedback effect that also occurred with miR-145 and its target gene, OCT4[42]. MiR-30c was previously reported to play a suppression role in tumour cell proliferation, metastasis and drug resistance by targeting BCL9[19], TWF1, vimentin[21] and KRAS[20]. In this study, we confirmed that miR-30c-MTA-1 signalling pathway represents a functional mechanism by which miR-30c suppresses EC. However, miRs usually work in the regulation of multiple targets and we could not tell that there is no other signalling pathway working by miR-30c in EC.

Currently, the regulation of miRNAs is a topic that has garnered increasing attention. Onconase[43], Lysophosphatidic acid (LPA)[19], and the EGF and MET receptors[24] have been reported to modulate the expression of miR-30c. Considering the differential expression of miR-30c between the ER-positive and ER-negative EC cell lines used in our study and the relationship between EC and E_2_, we chose to examine E_2_ as a candidate regulator of miR-30c expression.

In EC, miR-206[44] and the Let-7 family of miRNAs[9] correlate with E_2_ and ER either by targeting ERα or by being subject to modulation by E_2_. The modulation of miRNAs by E_2_-ER has been definitively demonstrated[45]. Yamagata et al[6] indicated that ERα, not ERβ, that inhibited the maturation of miRNAs by preventing the pri-miRNA-to-pre-miRNA conversion. This interaction occurs at a posttranscriptional level through their association with Drosha and p68/p72, which can be activated by E_2_. However, even in the absence of E_2_, a physical association between ERα and Drosha still occurs, possibly accounting for the suppression of miR-30c in the Ishikawa cells compared with the HEC-1-B cells that we observed in this study. Another recent study suggested that ERα suppressed miR-140 expression at the transcriptional level by binding to a specific promoter element (-79/-50) of miR-140[10]. Except for ERα, ERβ[14] and Dicer[13] were also reported to be relevant in terms of E_2_ in the regulation of miRNAs. Nevertheless, all of these results were derived from breast MCF-7 cells. Thus, we suggest that further mechanistic studies in EC cells are wanted.

Our study showed that E_2_ negatively regulated miR-30c and induced its target gene, MTA-1, in EC cells, a regulatory effect that was also exhibited by miR-140 and its target gene, SOX2, in breast cancer cells[10]. The induction of MTA-1 by E_2_ in EC cells might be attributed to a decrease in miR-30c or to direct stimulation by E_2_, both of which require further studies.

In contrast, a study has shown that miR-30c is up-regulated by E_2_ in ER-positive breast cancer MCF-7cells[13], demonstrating the different modulatory mechanisms by which E_2_ regulates miR-30c in different cancer cells. We considered that E_2_ functioned in an ER-dependent way and at transcriptional level in Ishikawa cells, because the decrease of miR-30c expression was reverted by ICI182780 and both pri-miR-30c and pre-miR-30c expression were repressed by E_2_ treatment. However, we could not exclude other possible regulatory mechanisms, such as the prevention of miRNAs maturation.

Unexpectedly, E_2_ repressed the expression of miR-30c in ER-negative HEC-1-B cells, which, to our knowledge has not yet been reported. These results imply that E_2_ can also function in an ER-independent manner, which has previously been observed in EC HEC-1-A cells[46] and in a mice model[47]. The reduction of pre-miR-30c, but not of pri-miR-30c, implies that E_2_ might influence the maturation of miR-30c in ER-independent way in HEC-1-B cells. However, little is known about ER-independent miRNA regulatory mechanisms. Further investigation on this topic might significantly contribute to our knowledge of the regulation of miRNAs, as well as of their numerous target genes.

In summary, this study indicates that miR-30c acts as a tumour suppressor by targeting MTA-1 in EC. Moreover, we find that miR-30c is negatively regulated by E_2_, shedding new light on an E_2_-ER-independent miRNA regulatory mechanism. Although there are some limitations to our study, such as the few numbers of EC samples and cell lines, the time and concentrations of E_2_ treatment, the lack of vivo studies, we still hope that our efforts will promote the continued investigation of the regulation of miR-30c and its role as a potential biomarker as well as a novel therapeutic target of EC, to demonstrate that miRNA-based clinical therapies are feasible options in the future.

## References

[1] Sorbe B (2012) Predictive and prognostic factors in definition of risk groups in endometrial carcinoma. ISRN Obstet Gynecol 2012: 325790. 23209924 10.5402/2012/325790 PMC3504391

[2] Kloosterman WP, Plasterk RH (2006) The diverse functions of microRNAs in animal development and disease. Dev Cell 11: 441–450. 17011485 10.1016/j.devcel.2006.09.009

[3] Ambros V (2004) The functions of animal microRNAs. Nature 431: 350–355. 15372042 10.1038/nature02871

[4] Sacco LD, Masotti A (2012) Recent Insights and Novel Bioinformatics Tools to Understand the Role of MicroRNAs Binding to 5′ Untranslated Region. Int J Mol Sci 14: 480–495. 23271365 10.3390/ijms14010480 PMC3565276

[5] Duursma AM, Kedde M, Schrier M, le Sage C, Agami R (2008) miR-148 targets human DNMT3b protein coding region. RNA 14: 872–877. 18367714 10.1261/rna.972008 PMC2327368

[6] Yamagata K, Fujiyama S, Ito S, Ueda T, Murata T, et al (2009) Maturation of microRNA is hormonally regulated by a nuclear receptor. Mol Cell 36: 340–347. 19854141 10.1016/j.molcel.2009.08.017

[7] Nothnick WB, Healy C (2010) Estrogen induces distinct patterns of microRNA expression within the mouse uterus. Reprod Sci 17: 987–994. 20720260 10.1177/1933719110377472 PMC4086356

[8] Pan Q, Luo X, Toloubeydokhti T, Chegini N (2007) The expression profile of micro-RNA in endometrium and endometriosis and the influence of ovarian steroids on their expression. Mol Hum Reprod 13: 797–806. 17766684 10.1093/molehr/gam063

[9] Zhang R, He Y, Zhang X, Xing B, Sheng Y, et al (2012) Estrogen receptor-regulated microRNAs contribute to the BCL2/BAX imbalance in endometrial adenocarcinoma and precancerous lesions. Cancer Lett 314: 155–165. 22014978 10.1016/j.canlet.2011.09.027

[10] Zhang Y, Eades G, Yao Y, Li Q, Zhou Q (2012) Estrogen receptor alpha signaling regulates breast tumor-initiating cells by down-regulating miR-140 which targets the transcription factor SOX2. J Biol Chem 287: 41514–41522. 23060440 10.1074/jbc.M112.404871 PMC3510847

[11] Masuda M, Miki Y, Hata S, Takagi K, Sakurai M, et al (2012) An induction of microRNA, miR-7 through estrogen treatment in breast carcinoma. Journal of Translational Medicine 10: S2. 23227519 10.1186/1479-5876-10-S1-S2 PMC3445861

[12] Wickramasinghe NS, Manavalan TT, Dougherty SM, Riggs KA, Li Y, et al (2009) Estradiol downregulates miR-21 expression and increases miR-21 target gene expression in MCF-7 breast cancer cells. Nucleic Acids Res 37: 2584–2595. 19264808 10.1093/nar/gkp117 PMC2677875

[13] Bhat-Nakshatri P, Wang G, Collins NR, Thomson MJ, Geistlinger TR, et al (2009) Estradiol-regulated microRNAs control estradiol response in breast cancer cells. Nucleic Acids Res 37: 4850–4861. 19528081 10.1093/nar/gkp500 PMC2724297

[14] He YQ, Sheng JQ, Ling XL, Fu L, Jin P, et al (2012) Estradiol regulates miR-135b and mismatch repair gene expressions via estrogen receptor-beta in colorectal cells. Exp Mol Med 44: 723–732. 23143558 10.3858/emm.2012.44.12.079 PMC3538979

[15] Zhao J, Imbrie GA, Baur WE, Iyer LK, Aronovitz MJ, et al (2013) Estrogen Receptor-Mediated Regulation of MicroRNA Inhibits Proliferation of Vascular Smooth Muscle Cells. Arterioscler Thromb Vasc Biol 33: 257–265. 23175673 10.1161/ATVBAHA.112.300200 PMC3780598

[16] Boren T, Xiong Y, Hakam A, Wenham R, Apte S, et al (2008) MicroRNAs and their target messenger RNAs associated with endometrial carcinogenesis. Gynecol Oncol 110: 206–215. 18499237 10.1016/j.ygyno.2008.03.023

[17] Zhou H, Xu X, Xun Q, Yu D, Ling J, et al (2012) microRNA-30c negatively regulates endometrial cancer cells by targeting metastasis-associated gene-1. Oncol Rep 27: 807–812. 22139444 10.3892/or.2011.1574

[18] Lee H, Park CS, Deftereos G, Morihara J, Stern JE, et al (2012) MicroRNA expression in ovarian carcinoma and its correlation with clinicopathological features. World J Surg Oncol 10: 174. 22925189 10.1186/1477-7819-10-174 PMC3449188

[19] Jia W, Eneh JO, Ratnaparkhe S, Altman MK, Murph MM (2011) MicroRNA-30c-2* expressed in ovarian cancer cells suppresses growth factor-induced cellular proliferation and downregulates the oncogene BCL9. Mol Cancer Res 9: 1732–1745. 22024689 10.1158/1541-7786.MCR-11-0245

[20] Tanic M, Yanowsky K, Rodriguez-Antona C, Andres R, Marquez-Rodas I, et al (2012) Deregulated miRNAs in hereditary breast cancer revealed a role for miR-30c in regulating KRAS oncogene. PLoS One 7: e38847. 22701724 10.1371/journal.pone.0038847 PMC3372467

[21] Bockhorn J, Yee K, Chang YF, Prat A, Huo D, et al (2013) MicroRNA-30c targets cytoskeleton genes involved in breast cancer cell invasion. Breast Cancer Res Treat 137: 373–382. 23224145 10.1007/s10549-012-2346-4 PMC3583223

[22] Rodriguez-Gonzalez FG, Sieuwerts AM, Smid M, Look MP, Meijer-van Gelder ME, et al (2011) MicroRNA-30c expression level is an independent predictor of clinical benefit of endocrine therapy in advanced estrogen receptor positive breast cancer. Breast Cancer Res Treat 127: 43–51. 20490652 10.1007/s10549-010-0940-x

[23] Li S, Yang C, Zhai L, Zhang W, Yu J, et al (2012) Deep sequencing reveals small RNA characterization of invasive micropapillary carcinomas of the breast. Breast Cancer Res Treat 136: 77–87. 22976804 10.1007/s10549-012-2166-6

[24] Garofalo M, Romano G, Di Leva G, Nuovo G, Jeon YJ, et al (2012) EGFR and MET receptor tyrosine kinase-altered microRNA expression induces tumorigenesis and gefitinib resistance in lung cancers. Nat Med 18: 74–82. 10.1038/nm.2577 PMC3467100 22157681

[25] Heinzelmann J, Henning B, Sanjmyatav J, Posorski N, Steiner T, et al (2011) Specific miRNA signatures are associated with metastasis and poor prognosis in clear cell renal cell carcinoma. World J Urol 29: 367–373. 21229250 10.1007/s00345-010-0633-4

[26] Ueda T, Volinia S, Okumura H, Shimizu M, Taccioli C, et al (2010) Relation between microRNA expression and progression and prognosis of gastric cancer: a microRNA expression analysis. Lancet Oncol 11: 136–146. 20022810 10.1016/S1470-2045(09)70343-2 PMC4299826

[27] Wang G, Zhang H, He H, Tong W, Wang B, et al (2010) Up-regulation of microRNA in bladder tumor tissue is not common. Int Urol Nephrol 42: 95–102. 19475496 10.1007/s11255-009-9584-3

[28] Guo J, Dong Q, Fang Z, Chen X, Lu H, et al (2010) Identification of miRNAs that are associated with tumor metastasis in neuroblastoma. Cancer Biol Ther 9: 446–452. 20147780 10.4161/cbt.9.6.10894

[29] Busacca S, Germano S, De Cecco L, Rinaldi M, Comoglio F, et al (2010) MicroRNA signature of malignant mesothelioma with potential diagnostic and prognostic implications. Am J Respir Cell Mol Biol 42: 312–319. 19502386 10.1165/rcmb.2009-0060OC

[30] Hummel R, Hussey DJ, Haier J (2010) MicroRNAs: predictors and modifiers of chemo- and radiotherapy in different tumour types. Eur J Cancer 46: 298–311. 19948396 10.1016/j.ejca.2009.10.027

[31] Bridge G, Monteiro R, Henderson S, Emuss V, Lagos D, et al (2012) The microRNA-30 family targets DLL4 to modulate endothelial cell behavior during angiogenesis. Blood 120: 5063–5072. 23086751 10.1182/blood-2012-04-423004

[32] Li XH, Ha CT, Fu D, Xiao M (2012) Micro-RNA30c negatively regulates REDD1 expression in human hematopoietic and osteoblast cells after gamma-irradiation. PLoS One 7: e48700. 23144934 10.1371/journal.pone.0048700 PMC3492427

[33] Patel N, Tahara SM, Malik P, Kalra VK (2011) Involvement of miR-30c and miR-301a in immediate induction of plasminogen activator inhibitor-1 by placental growth factor in human pulmonary endothelial cells. Biochem J 434: 473–482. 21175428 10.1042/BJ20101585 PMC3078570

[34] Duisters RF, Tijsen AJ, Schroen B, Leenders JJ, Lentink V, et al. (2009) miR-133 and miR-30 regulate connective tissue growth factor: implications for a role of microRNAs in myocardial matrix remodeling. Circ Res 104: : 170–178, 176p following 178. 10.1161/CIRCRESAHA.108.182535 19096030

[35] Joglekar MV, Patil D, Joglekar VM, Rao GV, Reddy DN, et al (2009) The miR-30 family microRNAs confer epithelial phenotype to human pancreatic cells. Islets 1: 137–147. 21099261 10.4161/isl.1.2.9578

[36] Li J, Donath S, Li Y, Qin D, Prabhakar BS, et al (2010) miR-30 regulates mitochondrial fission through targeting p53 and the dynamin-related protein-1 pathway. PLoS Genet 6: e1000795. 20062521 10.1371/journal.pgen.1000795 PMC2793031

[37] Balasenthil S, Broaddus RR, Kumar R (2006) Expression of metastasis-associated protein 1 (MTA1) in benign endometrium and endometrial adenocarcinomas. Hum Pathol 37: 656–661. 16733204 10.1016/j.humpath.2006.01.024

[38] Wang H, Fan L, Wei J, Weng Y, Zhou L, et al (2012) Akt mediates metastasis-associated gene 1 (MTA1) regulating the expression of E-cadherin and promoting the invasiveness of prostate cancer cells. PLoS One 7: e46888. 23227138 10.1371/journal.pone.0046888 PMC3515600

[39] Du B, Yang ZY, Zhong XY, Fang M, Yan YR, et al (2011) Metastasis-associated protein 1 induces VEGF-C and facilitates lymphangiogenesis in colorectal cancer. World J Gastroenterol 17: 1219–1226. 21448429 10.3748/wjg.v17.i9.1219 PMC3063917

[40] Eitan R, Kushnir M, Lithwick-Yanai G, David MB, Hoshen M, et al (2009) Tumor microRNA expression patterns associated with resistance to platinum based chemotherapy and survival in ovarian cancer patients. Gynecol Oncol 114: 253–259. 19446316 10.1016/j.ygyno.2009.04.024

[41] Sorrentino A, Liu CG, Addario A, Peschle C, Scambia G, et al (2008) Role of microRNAs in drug-resistant ovarian cancer cells. Gynecol Oncol 111: 478–486. 18823650 10.1016/j.ygyno.2008.08.017

[42] Wu Y, Liu S, Xin H, Jiang J, Younglai E, et al (2011) Up-regulation of microRNA-145 promotes differentiation by repressing OCT4 in human endometrial adenocarcinoma cells. Cancer 117: 3989–3998. 21365617 10.1002/cncr.25944

[43] Goparaju CM, Blasberg JD, Volinia S, Palatini J, Ivanov S, et al (2011) Onconase mediated NFKbeta downregulation in malignant pleural mesothelioma. Oncogene 30: 2767–2777. 21317924 10.1038/onc.2010.643 PMC3118086

[44] Chen X, Yan Q, Li S, Zhou L, Yang H, et al (2012) Expression of the tumor suppressor miR-206 is associated with cellular proliferative inhibition and impairs invasion in ERalpha-positive endometrioid adenocarcinoma. Cancer Lett 314: 41–53. 21983130 10.1016/j.canlet.2011.09.014

[45] Klinge CM (2012) miRNAs and estrogen action. Trends Endocrinol Metab 23: 223–233. 22503553 10.1016/j.tem.2012.03.002 PMC3348384

[46] Charnock-Jones DS, Sharkey AM, Rajput-Williams J, Burch D, Schofield JP, et al (1993) Identification and localization of alternately spliced mRNAs for vascular endothelial growth factor in human uterus and estrogen regulation in endometrial carcinoma cell lines. Biol Reprod 48: 1120–1128. 8481475 10.1095/biolreprod48.5.1120

[47] Karas RH, Schulten H, Pare G, Aronovitz MJ, Ohlsson C, et al (2001) Effects of estrogen on the vascular injury response in estrogen receptor alpha, beta (double) knockout mice. Circ Res 89: 534–539. 11557741 10.1161/hh1801.097239

